# The pear genomics database (PGDB): a comprehensive multi-omics research platform for *Pyrus* spp.

**DOI:** 10.1186/s12870-023-04406-5

**Published:** 2023-09-15

**Authors:** Shulin Chen, Manyi Sun, Shaozhuo Xu, Cheng Xue, Shuwei Wei, Pengfei Zheng, Kaidi Gu, Zhiwen Qiao, Zhiying Liu, Mingyue Zhang, Jun Wu

**Affiliations:** 1 College of Horticulture Science and Engineering, Shandong Agricultural University, Tai’an, 271018 Shandong China; 2 College of Horticulture, National Key Laboratory of Crop Genetics & Germplasm Enhancement and Utilization, Nanjing Agricultural University, Nanjing, 210095 Jiangsu China; 3 Shandong Institute of Pomology, Tai’an, 271000 China

**Keywords:** Pear, Database, Genome, Transcriptome, Re-sequencing

## Abstract

**Background:**

Pears are among the most important temperate fruit trees in the world, with significant research efforts increasing over the last years. However, available omics data for pear cannot be easily and quickly retrieved to enable further studies using these biological data.

**Description:**

Here, we present a publicly accessible multi-omics *pear* resource platform, the Pear Genomics Database (PGDB). We collected and collated data on genomic sequences, genome structure, functional annotation, transcription factor predictions, comparative genomics, and transcriptomics. We provide user-friendly functional modules to facilitate querying, browsing and usage of these data. The platform also includes basic and useful tools, including JBrowse, BLAST, phylogenetic tree building, and additional resources providing the possibility for bulk data download and quick usage guide services.

**Conclusions:**

The Pear Genomics Database (PGDB, http://pyrusgdb.sdau.edu.cn) is an online data analysis and query resource that integrates comprehensive multi-omics data for pear. This database is equipped with user-friendly interactive functional modules and data visualization tools, and constitutes a convenient platform for integrated research on pear.

**Supplementary Information:**

The online version contains supplementary material available at 10.1186/s12870-023-04406-5.

## Background

Pear is a member of the *Rosaceae* family and the *Amygdaloideae* subfamily [1], and one of the most important temperate fruit trees globally, with a cultivation history spanning more than 3,000 years [2, 3]. At present, 22 species and 5,000 accessions of pear have been described, including 5 major domesticate species cultivated for the production of fruit, specifically *P. communis*, *P. pyrifolia*, *P. bretschneideri*, *P. ussuriensis*, and *P. sinkiangensis* [4].

Most cultivated pears are diploid (2n = 34), and the genome is highly heterozygous and contains several repetitive sequences. The genome of an important oriental pear variety ‘Dangshansuli’ (*P. bretschneideri*) was sequenced and assembled using the HiSeq Illumina technology combined with a BAC-by-BAC strategy [5]. After this, the western variety ‘Bartlett’ (*P. communis*) was sequenced with Roche’s 454 Sequencing Technology [6]. In recent years, several more pear reference genomes were published owing the rapid development of sequencing technologies [7–13]. These developments further led to the generation of a large number of transcriptome and population DNA re-sequencing data, allowing mining key genes responsible for important agronomic traits and studying the domestication history of pears [14, 15]. At present, pear genome and resequencing data have been collected in the Rosaceae Genome database GDR, but transcriptome data is lacking. Therefore, there is an urgent need for a database that can effectively integrate, analyze and disseminate pear multiomics data, and provide a platform for researchers to quickly access and utilize these resources. These resources are already available for a variety of plants, such as bayberry and pineapple [16, 17]. Therefore, we integrated the advantages of the above-mentioned databases and constructed the Pear Genomics Database (PGDB). In this study, a total of nine genome sequences, 35 transcription group datasets, and re-sequencing data from 30 pear accessions were collected. We also included commonly used tools, such as BLAST, JBrowse, phylogenetic tree building in the PGDB which will facilitate the future development of pear functional genomics and molecular biology approaches.

### Database construction and content

**Fig. 1:**
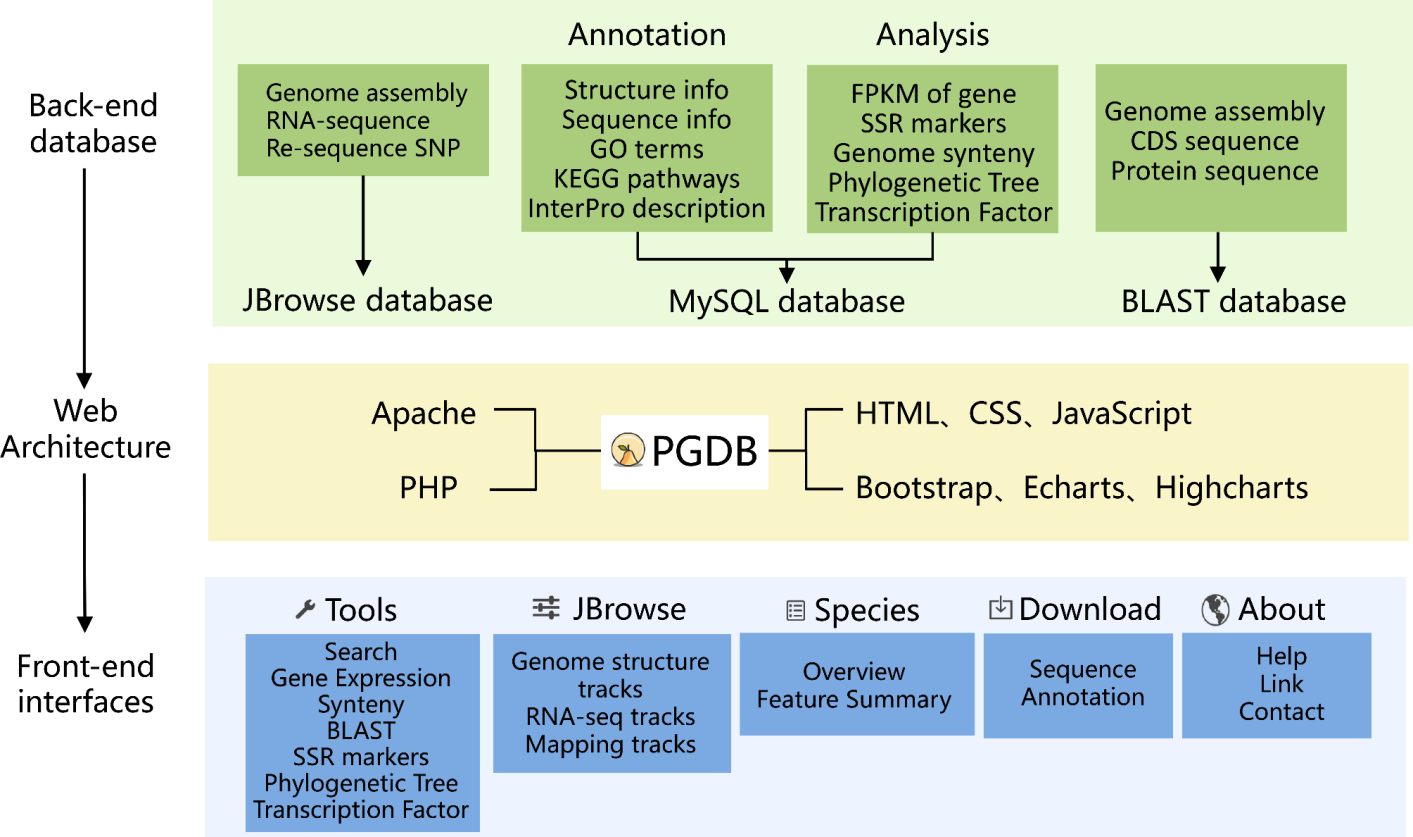
Overview of the PGDB website architecture

The PGDB collected and processed data on genome sequences, annotation, expression, synteny, and resequencing, which are stored in the MySQL database server (5.7.34). The web interface mainly uses the front-end framework Twitter Bootstrap based on HTML5 (HyperText Markup Language 5), CSS (Cascading Style Sheets) and JavaScript, and allows users to connect various levels of information, query the data and generate results. The data can be downloaded through a PHP protocol (7.4.21). The entire website was developed using the Web server software Apache (2.4.48), and implemented in the Linux (CentOS 7.6) operating system (Fig. 1).

### Genome assemblies and functional annotations

The PGBD collected information on 9 pear genomes, including ‘Dangshansuli’ v.1.0 (*P. bretschneideri*), ‘Dangshansuli’ v.1.1 (*P. bretschneideri*), ‘Cuiguan’ (*P. pyrifolia*), ‘Zhongai 1’ [(*P. ussuriensis* × *communis*) × spp.], ‘Shanxi Duli’ (*P. betulifolia*), ‘Nijisseiki’ (*P. pyrifolia*), ‘Bartlett’ v1.0 (*P. communis*), ‘Bartlett’ v2.0 (*P. communis*) and ‘d’Anjou’ (*P. communis*) (Additional file 1: Table S1). We kept the ID of each gene in the database consistent with the gene ID available in the original GFF annotation file. Gene Ontology (GO) [18] and InterPro [19] annotations were performed using InterProScan (v5.53-87.0) [20]. Kyoto Encyclopedia of Genes and Genomes (KEGG) pathways were annotated with the bi-directional best hit (BBH) method from KAAS [21] using 40 plant species as reference (Additional file 2: Table S2).

### Transcription factors

Transcription factors (TFs) and transcriptional regulators (TRs) modulate the expression of target genes, which in turn are involved in several important life processes, such as growth and development, secondary metabolism and abiotic stress responses [22, 23]. We used the iTAK program for TF and TR predictions, a software based on a set of consensus domain assignment rules [24]. We detected TFs and TRs from the aforementioned 9 pear genomes, i.e., ‘Dangshansuli’ v1.0 (2433, 563), ‘Cuiguan’ (2705, 616), ‘Shanxi Duli’ (2670, 628), ‘Zhongai 1’ (2409, 570), ‘Bartlett’ v1.0 (2541, 624), ‘Bartlett’ v2.0 (1910, 481), ‘Nijisseiki’ (2544, 625), ‘d’Anjou’ (2666,703), ‘Dangshansuli’ v1.1 (3517,475) (Additional file 3: Table S3), and found that the most numerous were the MYB and NAC families. These families include widely known key factors regulating development and stress responses [25, 26].

### Synteny data

We identified synteny blocks and homologous gene pairs from 9 pear genome data. The protein sequences were aligned against each other and themselves using BLASTP (E-value ≤ 1e–10). The MCScanX [27] software was then employed with default parameters to determine the synteny blocks and homologous gene pairs from the BLASTP results.

### Marker data

The Krait tool [28] was used to mine simple sequence repeat (SSR) resources in nine pear genome data. A total of 386,779 SSR markers were identified and divided into five categories, namely dinucleotides to hexanucleotides, with the minimum number of repeats of 6,5,4,4,4 for each SSR type. Primer3 software (58) [29] implemented in Krait tool was used to design SSR primers. The specific parameters are: the size range of polymerase chain reaction (PCR) product is 100–300 bp, the length of primer is 20–25 bases, the best is 22 bases, the best annealing temperature is 50–60 °C, the GC content is 40–60%, the best is 50%. Retain the default values for other parameters. In addition, 579 pairs of SSR markers were collected from reported literatures [30–35].

### Transcriptomic data

The PGDB included transcriptomic data from seven key stages of fruit development on the following 5 cultivars: ‘Hosui’ (*P. pyrifolia*), ‘Yali’ (*P. bretschneideri*), ‘Kuerlexiangli’ (*P. sinkiangensis*), ‘Nanguoli’ (*P. ussuriensis*) and ‘Starkrimson’ (*P. communis*) [36]. The RNA-seq reads were mapped to the reference genome using software of SOAPaligner [37]. Transcription abundance was quantified by in-house perl scripts using the method of mapped sequence reads per million kilobytes per exon (RPKM). In addition, the results were changed to bigwig format using deepTools [38] software and placed in JBrowse.

### Utility

#### Database content

The homepage of the PGDB database is mainly composed of three parts. The top navigation bar is a fast link entry of each module, including: ‘Tools’, ‘JBrowse’, ‘Species’, ‘Download’, and ‘About’. The middle part contains a brief introduction to the database and the fast link to the ‘Tools’ and ‘Species’ modules. The bottom portion includes the website’s launch date and other information.

### Available tools

#### Search

**Fig. 2:**
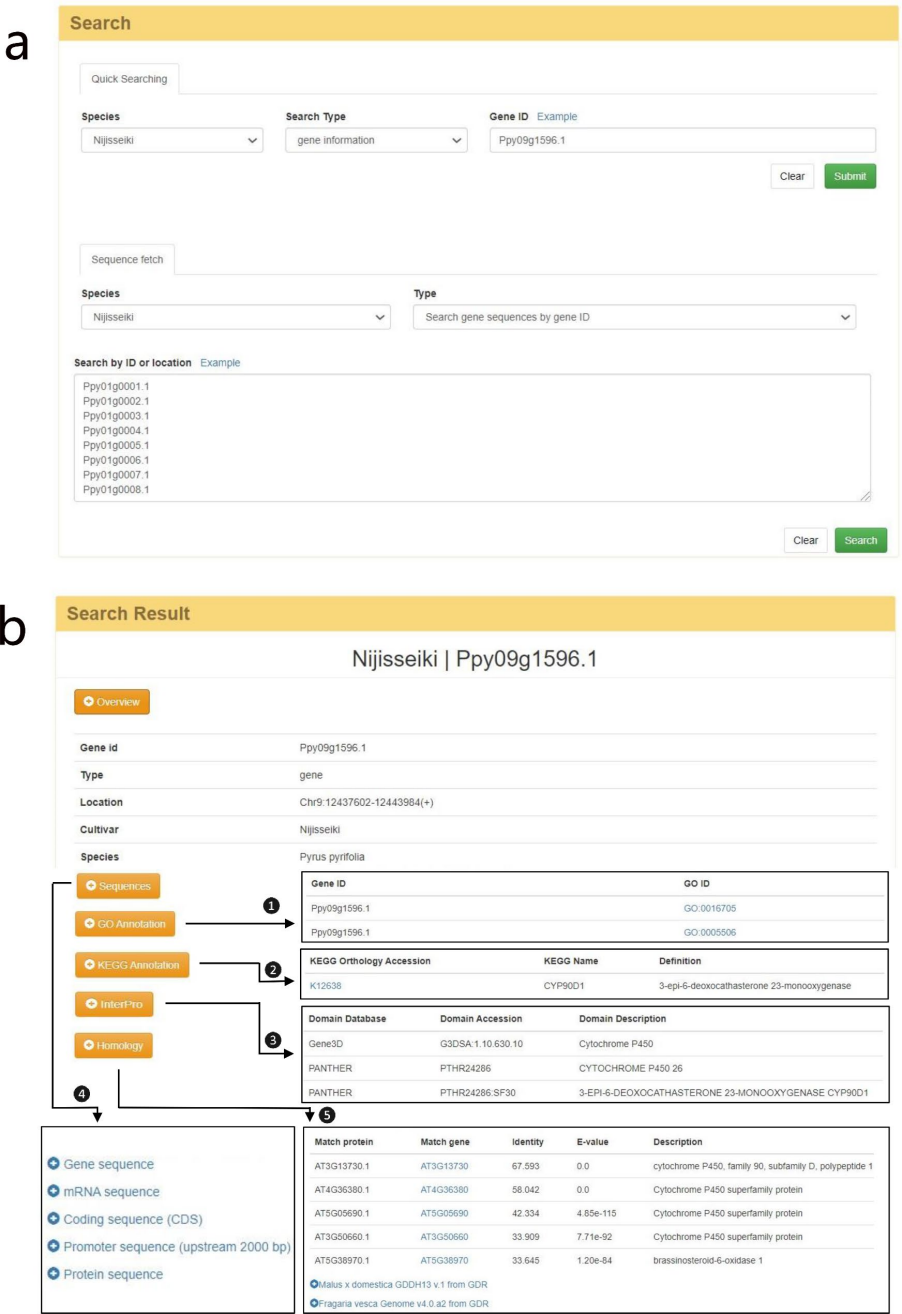
Search page of the PGDB. (**a**) Search for genetic information and sequences. (**b**) Genetic information search results, including gene details, GO ①, KEGG ② and InterPro ③ functional annotations, sequences ④, and homologous genes ⑤

The ‘Search’ page provides two retrieval modules (Fig. 2a). In the ‘Quick searching’ module, users can first search for detailed annotations on genes by simply selecting the cultivar genome and inputting the gene ID. The results page includes information on the sequences (including gene, CDS, and protein), functional annotations (GO, KEGG and InterPro) and the existence of homologous genes (Fig. 2b). Users can select which information should be displayed by clicking on different drop-down box options. In addition, users can also employ Bedtools [39] to retrieve genomic sequences by entering the reference genome coordinates. The results can be visualized online or downloaded for local storage. The ‘Sequence fetch’ module provides a batch search function for gene, CDS, and protein sequences.

### Gene expression

The ‘Gene Expression’ page provides a search function for genes with annotated RPKM values. Users can find this function in the navigation bar or the ‘Tools’ module in the middle section of the home page. The results are presented as line or bar charts drawn by Echarts [40] to display RPKM values at different development stages in pear fruits. The query results support online browsing and downloading to facilitate researchers conducting in-depth analyses.

### Synteny

**Fig. 3:**
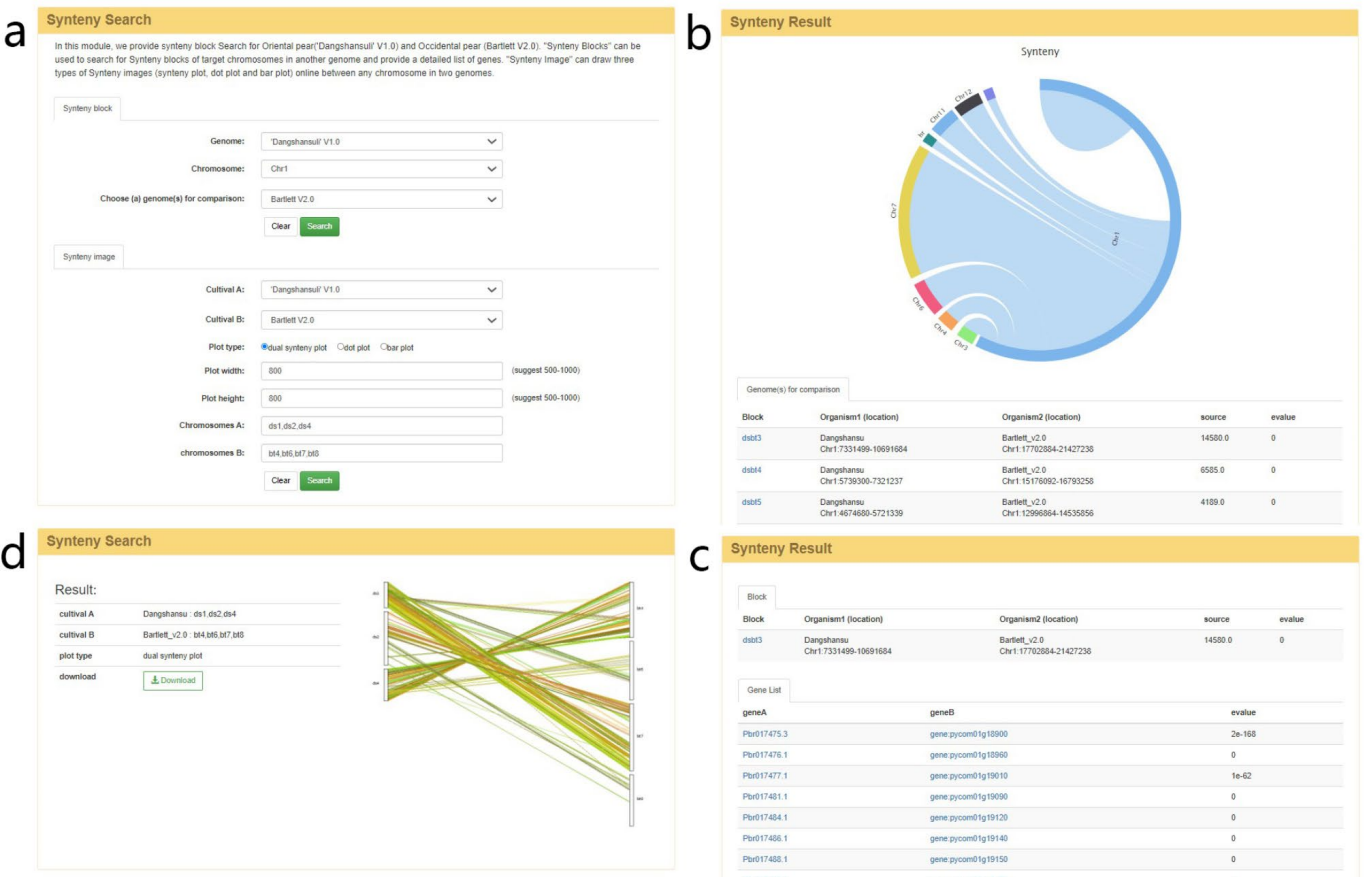
Synteny page of the PGDB. (**a**) Querying synteny blocks between genomes and drawing synteny images. (**b**) The synteny blocks in the query and compared chromosomes. (**c**) The genes contained in each synteny block. (**d**) The collinear image drawn online

In this page, comparative genomic information between different pear varieties is provided to facilitate quick retrieval of genomic collinearity and homologous gene pairs (Fig. 3a). In the ‘Synteny Block’ module, users can obtain synteny blocks by selecting the pear genome and chromosome of choice. The top half of the results page contains an image showing the quantitative relationship between synteny blocks of the query and compared genomes. This is implemented by HighCharts. The bottom half of the page provides complete synteny block information (block ID, location, source, e-value) in the form of a list (Fig. 3b). By clicking on different synteny blocks, users will be linked to detailed information on homologous gene pairs within synteny blocks (Fig. 3c). In the ‘Synteny Image’ module, synteny images can be constructed between the chromosomes of any two genomes, and downloaded for further study (Fig. 3d).

### BLAST

**Fig. 4:**
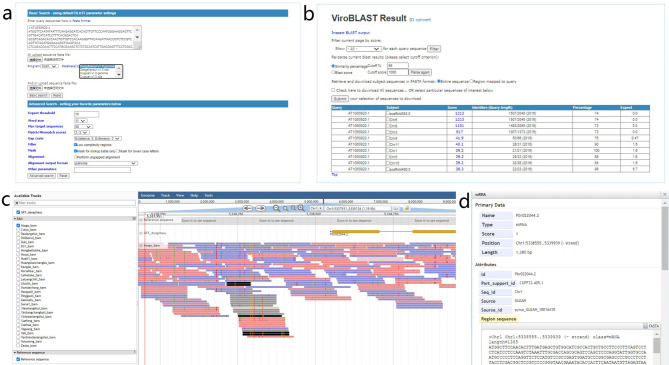
The BLAST and the JBrowse tools available in the PGDB. (**a**) BLAST page to search for regions of similarity between sequences. (**b**) The BLAST results page. (**c**) Visualization of genomic regions using the Genome browser. (**d**) Detailed information about a single region

This page provides a user-friendly BLAST tool for sequence alignment with ViroBlast [41]. Nucleotide and amino acid sequence similarity searches can be performed through a user-friendly input-output interface. We provide three types of query databases for genomic sequences, CDS sequences and protein sequences (Fig. 4a, b). Users can search the nucleotide sequence and the protein sequence databases by query sequences in BLASTN or BLASTX, and TBLASTN and BLASTP, respectively. In addition, users can choose TBLASTX to translate nucleotide sequences into protein sequences before comparison.

### SSR markers

PGDB provides a query page for two types of SSR markers based on genomic prediction and literature reports. Users search for molecular marker data by filling in SSR IDs or selecting special items. Users can submit the search criteria to obtain detailed information including variety, SSR ID, scaffold, motif, type, repeat, start, end, and length. In addition, for genomic SSR markers, detailed information related to primers can be obtained by clicking SSR ID, such as forward sequence, reverse sequence, Tm (temperature), GC content and product size, etc.

### Phylogenetic tree building

This page provides a simple and quick tool for constructing phylogenetic trees. Users can input FASTA formatted sequences, with alignment performed with MAFFT (V7.158) [42]. IQ-Tree, a stochastic algorithm to infer phylogenetic trees by maximum likelihood, is then used to assemble these sequences [43, 44]. Both the aligned sequence file and the NWK file containing the phylogenetic tree can be downloaded. Finally the Phylo.io [45] tool was used for the visual presentation of the phylogenetic trees.

### Transcription factor

This page provides a search function for predicted TF and TR families in the 9 pear genomes. The search form allows users to retrieve additional TF families by entering a specific gene ID or, instead, the family name for a complete list of genes in specific families. We also provide a list of 94 families at the bottom of the search page to serve as reference.

### Genome browser

The genome browser is an important tool for visualization of high-throughput sequencing data. JBrowse [46] is a genome browser based on HTML5 and JavaScript, which contains a fully dynamic AJAX interface. We collected genome and annotation information for 9 pear varieties, as well as genome re-sequencing data for 30 pear cultivars, which were mapped to the ‘Dangshansuli’ v1.0 genome [1, 47, 48]. In addition, we mapped transcriptome data from five pear cultivars of seven stages to nine pear reference genomes. These data can all be viewed in JBrowse. On the left-hand side of the genome browser, the ‘Available Tracks’ option provides all displayable file options. After choosing which files to display, the information will appear on a window located in the right-hand side (Fig. 4c). Clicking on the different parts of the sequences will display detailed data information and allows users to browse gene sequences, structure and annotations (Fig. 4d).

### Other options

The ‘Species’ page contains a brief introduction to the 9 pear genomes available and provides links to the relevant literature. The ‘About’ module contains three parts: the ‘Download’ page allows users to download genomic information, including FASTA files of the genome assembly, gene, CDS, and protein sequences, and gene structure data in the GFF format. The ‘Link’ page provides quick links to other plant-related databases and resources. The ‘Contact’ option allows users to contact the administrators of the PGDB.

## Conclusion

PGDB currently includes genomic, transcriptomic and re-sequencing data for pear, which can be displayed through a user-friendly platform that is functionally practical. This can help researchers quickly retrieving, browsing and analyzing multi-omics data and promote in-depth studies and development of pear omics.

### Electronic supplementary material

Below is the link to the electronic supplementary material.

Table S1: Profile of the 8 pear genomes available in the PGDB

Table S2: Functional annotations of pear genomes, including GO terms, KEGG pathways and InterPro terms

Table S3: Transcription factor and regulator gene families in 9 pear genomes

## Data Availability

All original RNA-seq data (BioProject PRJNA309745) and re-sequencing data (BioProject PRJNA563813, PRJNA782471, PRJNA381668) are available at the National Center for Biotechnology Information (NCBI) database. This database can be accessed for free at http://pyrusgdb.sdau.edu.cn.

## Abbreviations

BAC-by-BAC: Bacterial artificial chromosome
MySQL: My’s Structured Query Language
HTML5: HyperText Markup Language, version 5
CSS: Cascading Style Sheets
PHP: PHP Hypertext Preprocessor
AJAX: Asynchronous JavaScript and XML
GFF: Generic Feature Format
GO: Gene Ontology
KEGG: Kyoto Encyclopedia of Genes and Genomes
GDR: Genome Database for Rosaceae
KAAS: KEGG Automatic Annotation Server
CDS: Coding DNA Sequence
TFs: Transcription factors
TRs: Transcriptional regulators
RPKM: Reads Per Kilobase per Million mapped reads
BLAST: Basic Local Alignment Search Tool
NWK: Newick
AJAX: Asynchronous JavaScript and XML
NCBI: National Center for Biotechnology Information

## References

[1] Wu J, Wang Y, Xu J, Korban SS, Fei Z, Tao S, Ming R, Tai S, Khan AM, Postman JD (2018). Diversification and independent domestication of asian and european pears. Genome Biol.

[2] Ferradini N, Lancioni H, Torricelli R, Russi L, Dalla Ragione I, Cardinali I, Marconi G, Gramaccia M, Concezzi L, Achilli A (2017). Characterization and phylogenetic analysis of ancient italian landraces of Pear. Front Plant Sci.

[3] Wu J, Li LT, Li M, Khan MA, Li XG, Chen H, Yin H, Zhang SL (2014). High-density genetic linkage map construction and identification of fruit-related QTLs in pear using SNP and SSR markers. J Exp Bot.

[4] Li J, Zhang M, Li X, Khan A, Kumar S, Allan AC, Lin-Wang K, Espley RV, Wang C, Wang R et al. Pear genetics: recent advances, new prospects, and a roadmap for the future. Hortic Res 2022, 9. 10.1093/hr/uhab040 PMC8778596 35031796

[5] Wu J, Wang Z, Shi Z, Zhang S, Ming R, Zhu S, Khan MA, Tao S, Korban SS, Wang H (2013). The genome of the pear (Pyrus bretschneideri Rehd). Genome Res.

[6] Chagne D, Crowhurst RN, Pindo M, Thrimawithana A, Deng C, Ireland H, Fiers M, Dzierzon H, Cestaro A, Fontana P et al. The Draft Genome Sequence of European Pear (Pyrus communis L. ‘Bartlett’). PLoS ONE 2014, 9(4). 10.1371/journal.pone.0092644 PMC3974708 24699266

[7] Shirasawa K, Itai A, Isobe S. Chromosome-scale genome assembly of Japanese pear (Pyrus pyrifolia) variety ‘Nijisseiki’. DNA Res 2021, 28(2). 10.1093/dnares/dsab001 PMC8092371 33638981

[8] Ou C, Wang F, Wang J, Li S, Zhang Y, Fang M, Ma L, Zhao Y, Jiang S (2019). A de novo genome assembly of the dwarfing pear rootstock Zhongai 1. Sci Data.

[9] Gao YH, Yang QS, Yan XH, Wu XY, Yang F, Li JZ, Wei J, Ni JB, Ahmad M, Bai SL et al. High-quality genome assembly of ‘Cuiguan’ pear (Pyrus pyrifolia) as a reference genome for identifying regulatory genes and epigenetic modifications responsible for bud dormancy. Hortic Res-England 2021, 8(1). 10.1038/s41438-021-00632-w PMC8408243 34465760

[10] Linsmith G, Rombauts S, Montanari S, Deng CH, Celton JM, Guerif P, Liu C, Lohaus R, Zurn JD, Cestaro A et al. Pseudo-chromosome-length genome assembly of a double haploid “Bartlett” pear (Pyrus communis L.). Gigascience 2019, 8(12). 10.1093/gigascience/giz138 PMC6901071 31816089

[11] Dong X, Wang Z, Tian L, Zhang Y, Qi D, Huo H, Xu J, Li Z, Liao R, Shi M (2020). De novo assembly of a wild pear (Pyrus betuleafolia) genome. Plant Biotechnol J.

[12] Zhang H, Wafula EK, Eilers J, Harkess AE, Ralph PE, Timilsena PR, dePamphilis CW, Waite JM, Honaas LA (2022). Building a foundation for gene family analysis in Rosaceae genomes with a novel workflow: a case study in Pyrus architecture genes. Front Plant Sci.

[13] Xue H, Wang S, Yao JL, Deng CH, Wang L, Su Y, Zhang H, Zhou H, Sun M, Li X (2018). Chromosome level high-density integrated genetic maps improve the Pyrus bretschneideri ‘DangshanSuli’ v1.0 genome. BMC Genomics.

[14] Li XL, Liu L, Ming ML, Hu HJ, Zhang MY, Fan J, Song BB, Zhang SL, Wu J (2019). Comparative transcriptomic analysis provides insight into the domestication and improvement of Pear (P. pyrifolia) Fruit. Plant Physiol.

[15] Song B, Li X, Cao B, Zhang M, Korban SS, Yu L, Yang W, Zhao K, Li J, Wu J (2022). An identical-by-descent segment harbors a 12-bp insertion determining fruit softening during domestication and speciation in Pyrus. BMC Biol.

[16] Ren HY, He YH, Qi XJ, Zheng XL, Zhang SW, Yu ZP, Hu FR. The bayberry database: a multiomic database for Myrica rubra, an important fruit tree with medicinal value. BMC Plant Biol 2021, 21(1). 10.1186/s12870-021-03232-x PMC8493685 34615485

[17] Xu HM, Yu QY, Shi Y, Hua XT, Tang HB, Yang L, Ming R, Zhang JS. PGD: Pineapple Genomics database. Hortic Res-England 2018, 5. 10.1038/s41438-018-0078-2 PMC6139296 30245835

[18] Gene Ontology C (2015). Gene Ontology Consortium: going forward. Nucleic Acids Res.

[19] Finn RD, Attwood TK, Babbitt PC, Bateman A, Bork P, Bridge AJ, Chang HY, Dosztanyi Z, El-Gebali S, Fraser M (2017). InterPro in 2017-beyond protein family and domain annotations. Nucleic Acids Res.

[20] Jones P, Binns D, Chang HY, Fraser M, Li W, McAnulla C, McWilliam H, Maslen J, Mitchell A, Nuka G (2014). InterProScan 5: genome-scale protein function classification. Bioinformatics.

[21] Moriya Y, Itoh M, Okuda S, Yoshizawa AC, Kanehisa M. KAAS: an automatic genome annotation and pathway reconstruction server. Nucleic Acids Res 2007, 35(Web Server issue):W182–185. 10.1093/nar/gkm321 PMC1933193 17526522

[22] Gong W, Shen YP, Ma LG, Pan Y, Du YL, Wang DH, Yang JY, Hu LD, Liu XF, Dong CX (2004). Genome-wide ORFeome cloning and analysis of Arabidopsis transcription factor genes. Plant Physiol.

[23] Manna M, Thakur T, Chirom O, Mandlik R, Deshmukh R, Salvi P (2021). Transcription factors as key molecular target to strengthen the drought stress tolerance in plants. Physiol Plant.

[24] Zheng Y, Jiao C, Sun H, Rosli HG, Pombo MA, Zhang P, Banf M, Dai X, Martin GB, Giovannoni JJ (2016). iTAK: a program for genome-wide prediction and classification of plant transcription factors, transcriptional regulators, and protein kinases. Mol Plant.

[25] Jensen MK, Kjaersgaard T, Nielsen MM, Galberg P, Petersen K, O’Shea C, Skriver K (2010). The Arabidopsis thaliana NAC transcription factor family: structure-function relationships and determinants of ANAC019 stress signalling. Biochem J.

[26] Dubos C, Stracke R, Grotewold E, Weisshaar B, Martin C, Lepiniec L (2010). MYB transcription factors in Arabidopsis. Trends Plant Sci.

[27] Wang Y, Tang H, Debarry JD, Tan X, Li J, Wang X, Lee TH, Jin H, Marler B, Guo H (2012). MCScanX: a toolkit for detection and evolutionary analysis of gene synteny and collinearity. Nucleic Acids Res.

[28] Du L, Zhang C, Liu Q, Zhang X, Yue B, Hancock J (2018). Krait: an ultrafast tool for genome-wide survey of microsatellites and primer design. Bioinformatics.

[29] Untergasser A, Cutcutache I, Koressaar T, Ye J, Faircloth BC, Remm M, Rozen SG (2012). Primer3–new capabilities and interfaces. Nucleic Acids Res.

[30] Chen H, Song Y, Li LT, Khan MA, Li XG, Korban SS, Wu J, Zhang SL (2015). Construction of a high-density simple sequence repeat Consensus Genetic Map for Pear (Pyrus spp). Plant Mol Biol Rep.

[31] Yamamoto T, Kimura T, Shoda M, Imai T, Saito T, Sawamura Y, Kotobuki K, Hayashi T, Matsuta N (2002). Genetic linkage maps constructed by using an interspecific cross between japanese and european pears. Theor Appl Genet.

[32] Yamamoto T, Kimura T, Shoda M, Ban Y, Hayashi T, Matsuta N. Development of microsatellite markers in the japanese pear (Pyrus pyrifolia Nakai). Mol Ecol Notes 2002(1):2.

[33] Inoue E, Matsuki Y, Anzai H, Evans K. Isolation and characterization of microsatellite markers in japanese pear (Pyrus pyrifolia Nakai). Molecular Ecology Notes; 2007.

[34] FERNÁNDEZ-FERNÁNDEZ F, Harvey NG, James CM (2010). Isolation and characterization of polymorphic microsatellite markers from european pear (Pyrus communis L). Mol Ecol Notes.

[35] Lei WANG, Long WANG, Hua-bai XUE, Xiu-gen LI, Jiang LI (2016). Construction of SSR genetic linkage map and comparison on Pears[J]. Scientia Agricultura Sinica.

[36] Zhang MY, Xue C, Xu L, Sun H, Qin MF, Zhang S, Wu J (2016). Distinct transcriptome profiles reveal gene expression patterns during fruit development and maturation in five main cultivated species of pear (Pyrus L). Sci Rep.

[37] Li R, Yu C, Li Y, Lam TW, Yiu SM, Kristiansen K, Wang J (2009). SOAP2: an improved ultrafast tool for short read alignment. Bioinformatics.

[38] Ramirez F, Dundar F, Diehl S, Gruning BA, Manke T. deepTools: a flexible platform for exploring deep-sequencing data. Nucleic Acids Res 2014, 42(Web Server issue):W187–191. 10.1093/nar/gku365 PMC4086134 24799436

[39] Quinlan AR, Hall IM (2010). BEDTools: a flexible suite of utilities for comparing genomic features. Bioinformatics.

[40] Li DQ, Mei HH, Shen Y, Su S, Zhang WL, Wang JT, Zu M, Chen W. ECharts: A declarative framework for rapid construction of web-based visualization (vol 2, pg 136, 2018). *Visual Informatics* 2021, 5(1):43–43.

[41] Deng W, Nickle DC, Learn GH, Maust B, Mullins JI (2007). ViroBLAST: a stand-alone BLAST web server for flexible queries of multiple databases and user’s datasets. Bioinformatics.

[42] Katoh K, Standley DM (2013). MAFFT multiple sequence alignment software version 7: improvements in performance and usability. Mol Biol Evol.

[43] Nguyen LT, Schmidt HA, von Haeseler A, Minh BQ (2015). IQ-TREE: a fast and effective stochastic algorithm for estimating maximum-likelihood phylogenies. Mol Biol Evol.

[44] Kalyaanamoorthy S, Minh BQ, Wong TKF, von Haeseler A, Jermiin LS (2017). ModelFinder: fast model selection for accurate phylogenetic estimates. Nat Methods.

[45] Robinson O, Dylus D, Dessimoz C (2016). Phylo.io: interactive viewing and comparison of large phylogenetic trees on the web. Mol Biol Evol.

[46] Buels R, Yao E, Diesh CM, Hayes RD, Munoz-Torres M, Helt G, Goodstein DM, Elsik CG, Lewis SE, Stein L (2016). JBrowse: a dynamic web platform for genome visualization and analysis. Genome Biol.

[47] Zhang MY, Xue C, Hu H, Li J, Xue Y, Wang R, Fan J, Zou C, Tao S, Qin M (2021). Genome-wide association studies provide insights into the genetic determination of fruit traits of pear. Nat Commun.

[48] Chen XN, Zhang MY, Sun MY, Liu YY, Li SN, Song BB, Li MY, Zhang SL, Wang RZ, Li JM et al. Genome-wide genetic diversity and IBD analysis reveals historic dissemination routes of pear in China. Tree Genet Genomes 2022, 18(1).

